# Lung cancer staging

**DOI:** 10.1186/1470-7330-15-S1-O20

**Published:** 2015-10-02

**Authors:** Stefan Diederich, Matthijs Oudkerk

**Affiliations:** 1 Department of Diagnostic and Interventional Radiology, Marien Hospital, 40479 Düsseldorf, Germany; 2 Department of Radiology, Faculty of Medical Sciences, University Medical Center, 9700 Groningen, The Netherlands

## 

During this interactive hands-on workshop the participants will view complete imaging examinations (CT of chest, abdomen, brain, MRI of brain, chest, adrenals, musculoskeletal system, PET-CT, bone scintigraphy etc.) on workstations in a clinical routine setting and discuss findings with two tutors.

Cases will include those with typical findings as well as pitfalls and problems.

Particular attention will be paid to the correct assessment of findings that alter the therapeutic concepts [1-3].
